# Cardiosomal microRNAs Are Essential in Post-Infarction Myofibroblast Phenoconversion

**DOI:** 10.3390/ijms21010201

**Published:** 2019-12-27

**Authors:** Marco B. Morelli, Jun Shu, Celestino Sardu, Alessandro Matarese, Gaetano Santulli

**Affiliations:** 1 Department of Medicine, Division of Cardiology and Department of Molecular Pharmacology, Fleischer Institute for Diabetes and Metabolism (FIDAM), Albert Einstein College of Medicine, Montefiore University Hospital, New York, NY 10461, USA; marco.morelli@einsteinmed.org (M.B.M.); jun.shu@einstein.yu.edu (J.S.); 2 Department of Medical, Surgical, Neurological, Metabolic and Aging Sciences, University of Campania “Luigi Vanvitelli”, 80100 Naples, Italy; celestino.sardu@unicampania.it; 3 Department of Pneumology and Oncology, AORN “Ospedale dei Colli”, 80100 Naples, Italy; alessandromatarese@yahoo.it; 4 Department of Advanced Biomedical Science, “Federico II” University, and International Translational Research and Medical Education Consortium (ITME), 80131 Naples, Italy

**Keywords:** cardiac ischemia, epigenetics, exosomes, extracellular vesicles, fibroblasts, inflammation, microRNA, myocardial infarction, myofibroblast activation

## Abstract

The inclusion of microRNAs (miRNAs) in extracellular microvesicles/exosomes (named cardiosomes when deriving from cardiomyocytes) allows their active transportation and ensures cell-cell communication. We hypothesize that cardiosomal miRNAs play a pivotal role in the activation of myofibroblasts following ischemic injury. Using a murine model of myocardial infarction (MI), we tested our hypothesis by measuring in isolated fibroblasts and cardiosomes the expression levels of a set of miRNAs, which are upregulated in cardiomyocytes post-MI and involved in myofibroblast phenoconversion. We found that miR-195 was significantly upregulated in cardiosomes and in fibroblasts isolated after MI compared with SHAM conditions. Moreover, primary isolated cardiac fibroblasts were activated both when incubated with cardiosomes isolated from ischemic cardiomyocytes and when cultured in conditioned medium of post-MI cardiomyocytes, whereas no significant effect was observed following incubation with cardiosomes or medium from sham cardiomyocytes. Taken together, our findings indicate for the first time that a cardiomyocyte-specific miRNA, transferred to fibroblasts in form of exosomal cargo, is crucial in the activation of myofibroblasts.

## 1. Introduction

Mounting evidence has reinforced the concept that cardiac fibroblasts are much more than simple homeostatic regulators of the extracellular matrix (ECM) turnover, being in fact integrally involved in all aspects of the repair and remodeling of the heart occurring after an ischemic injury [1,2,3,4]. Although cardiac fibroblasts have been less widely studied than cardiomyocytes, it is becoming increasingly apparent that these cells (and their differentiated phenotype, myofibroblasts) are integral to the development, normal function, and repair of the heart, and are the dominating cell type in the processes of cardiac remodeling and fibrosis [4,5,6,7].

Recently, microRNAs (miRNAs, miRs) have emerged as regulators of cell-cell communication and signaling mediators during both physiological and pathological processes [8,9,10,11,12]. miRNAs are short, non-coding nucleotides that repress the expression of target genes through mRNA degradation or translational repression [13]. An altered expression of miRNAs has been linked to cardiovascular disorders, including heart failure and left ventricular hypertrophy [14,15,16]. Moreover, non-coding RNAs and miRNAs have been implied in myofibroblasts activation [17]. The discovery of circulating extracellular miRNAs in body fluids indicates a new role for miRNAs as cell-cell signaling mediators [18,19]. Hence, miRNAs are actively transported, either by binding to RNA-binding proteins or by the inclusion in extracellular microvesicles/exosomes (named cardiosomes when derived from cardiomyocytes), allowing protection from ribonuclease-dependent degradation [20,21,22,23]. Extracellular vesicles incorporating miRNAs represent one of the most actively investigated areas in the cardiovascular field, being studied both as potential diagnostic and prognostic biomarkers in a number of cardiovascular pathologies and associated metabolic diseases [24,25,26,27,28,29].

Our hypothesis is that cardiosomal miRNAs play a key role in the activation of myofibroblasts following ischemic injury.

## 2. Results

### 2.1. Cardiac Ischemic Injury Causes Upregulation of miR-195 in Myofibroblasts

After myocardial infarction (MI), quiescent cardiac fibroblasts can acquire an activated phenotype, and a recognized marker of such activation is the expression of α-smooth muscle actin (α-SMA); such cells are commonly known as myofibroblasts [30,31,32]. Through means of bioinformatic studies we identified several cardiomyocyte-specific miRNAs that enhance α-SMA expression targeting its inhibitor SMAD7 (mothers against DPP homologs 7), and we focused on miR-195 inasmuch as it has such targets predicted both in human and murine genomes and is upregulated following cardiac ischemia [33]. 

**Figure 1:**
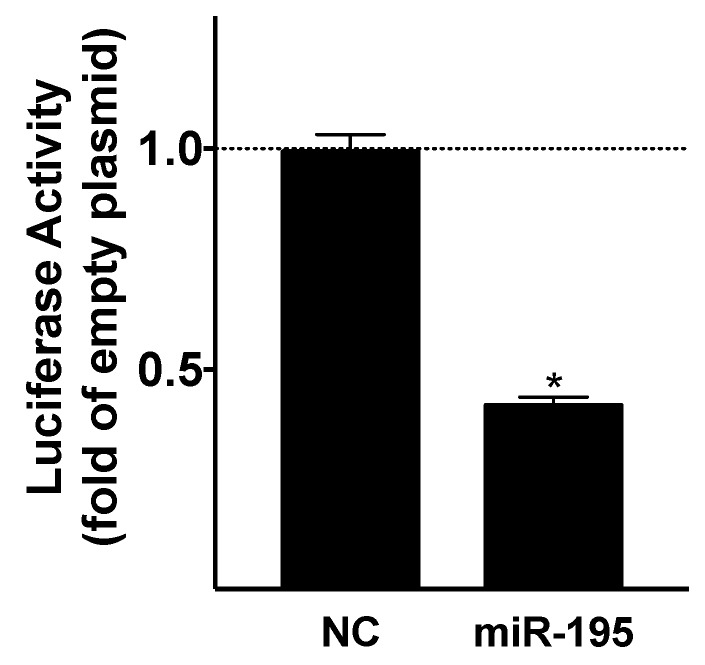
Identification of miR-195 as negative regulator of SMAD7. Luciferase activity was measured 48 h after transfection, and the values are shown as fold change of the luciferase activity with respect to the empty plasmid; cel-miR-39 mimic was used a negative control (NC). Mean ± S.E.M. of at least three independent experiments; * *p* < 0.05 vs. NC.

First, we biologically validated SMAD7 as an actual target of this miRNA by performing a luciferase assay (Figure 1). 

**Figure 2:**
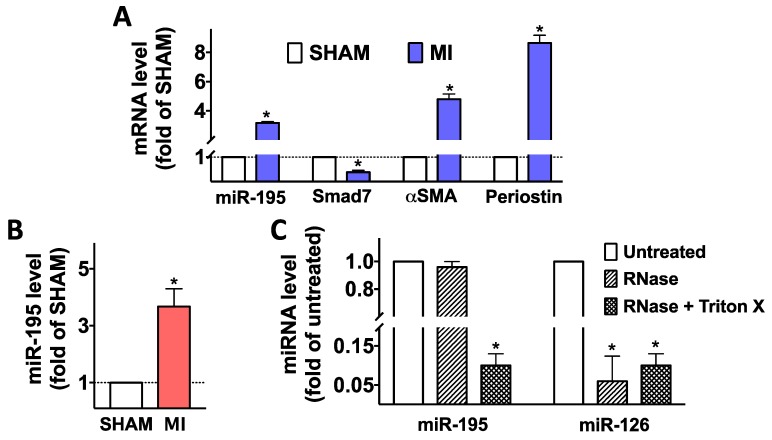
Upregulation of miR-195 post MI. This miRNA was upregulated in fibroblasts (**A**) as well as in exosomes obtained from cardiomyocytes (cardiosomes, **B**) isolated seven days after MI (*n* ≥ 6 mice/group); Smad7 mRNA was significantly down-regulated whereas αSMA and periostin, known markers of myofibroblast activation, were upregulated in fibroblasts post-MI (**A**). In panel **C**, cardiosome preparations were re-suspended in 300 mL PBS and spiked with 20 ρmol of a synthetic oligonucleotide corresponding to the mature sequence of miR-126 (exogenous miRNA used as control); samples were then treated or not with Triton X-100 (1%) and incubated with or without RNase A (0.5 U) and T1 (15 U) for 30 min at 37 °C before RNA extraction. Mean ± S.E.M. of at least three independent experiments; * *p* < 0.05 vs. SHAM (panels A, B) or untreated (panel C).

Then, we moved to an in vivo setting, using an established murine model of MI, achieved via permanent occlusion of the left anterior descending coronary artery [34]; myocardial infarction was confirmed by measuring Troponin I levels in serum 1 day after surgery (MI: 60.8 ± 13.2 ng/mL vs. SHAM: 1.9 ± 0.6 ng/mL; *p* < 0.001) as well as by echocardiography, assessed before euthanizing the mice in terms of left ventricular ejection fraction (MI: 42.3 ± 5.7% vs. SHAM: 78.1 ± 2.7%; *p* < 0.01 and left ventricular fractional shortening (MI: 22.8 ± 1.5 ng/mL vs. SHAM: 50.6 ± 1.9%; *p* < 0.01). We demonstrated via quantitative real time PCR (RT-qPCR) that miR-195 is upregulated in fibroblasts post-ischemia (Figure 2A) and is also contained in cardiosomes (Figure 2B). To ensure that this miRNA was actually confined inside cardiosomes, the samples were treated with RNase, showing that the level of miRNAs was not affected by RNase treatment, unless in presence of Triton X-100 (Figure 2C). The activation of myofibroblasts was confirmed by the increased expression of αSMA and periostin (Figure 2A).

### 2.2. Fibroblasts Are Activated Following the Incubation with Cardiosomes Isolated from Ischemic Cardiomyocytes

**Figure 3:**
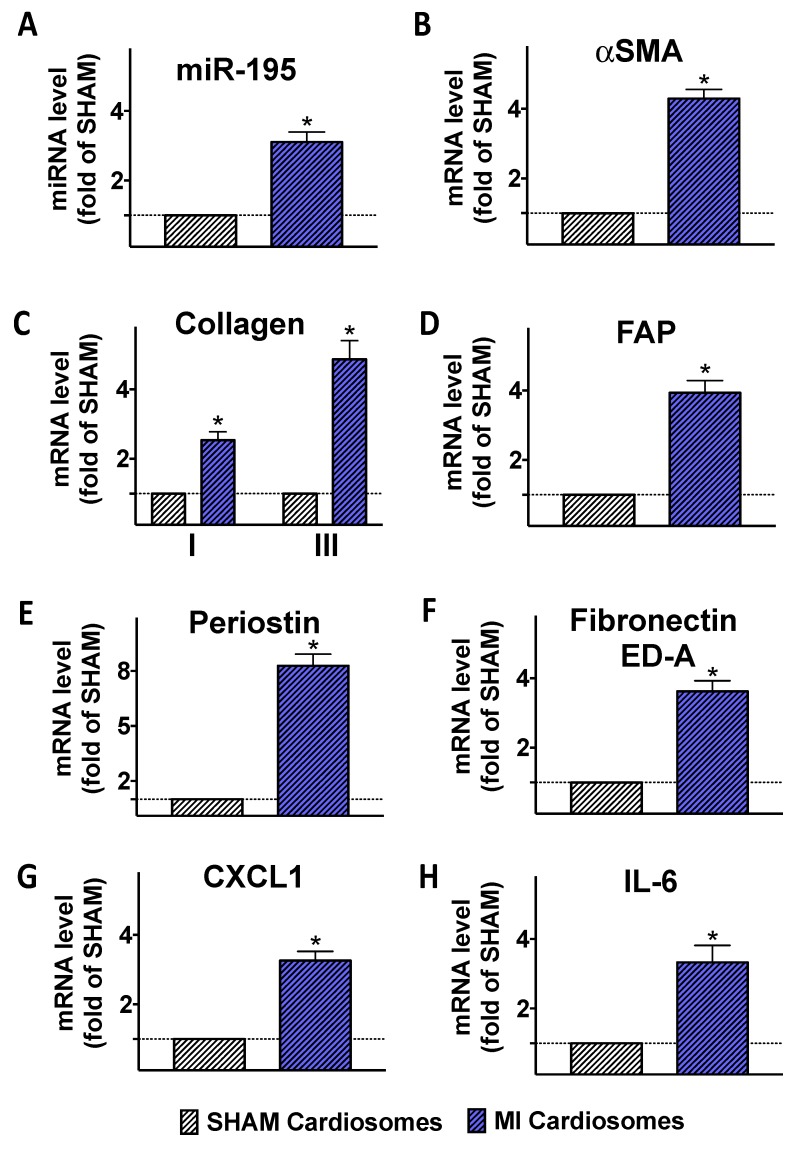
Effects of cardiosomes on fibroblast activation. Fibroblasts were incubated with cardiosomes obtained from cardiomyocytes isolated from SHAM and MI mice (*n* ≥ 6 mice/group) seven days post-surgery; such incubation induced an upregulation of miR-195 (**A**), αSMA (**B**), collagen I and III (**C**) fibroblast activation protein (FAP, **D**), periostin (**E**), fibronectin containing extra domain A (Fibronectin ED-A, **F**), chemokine (C-X-C motif) ligand 1 (CXCL1, **G**), interleukin-6 (IL-6, **H**). Mean ± S.E.M. of at least three independent experiments; * *p* < 0.05 vs. SHAM.

To confirm that the cardiosomal miRNA cargo was actually transferred to fibroblasts, we performed a series of ex vivo experiments: after isolating primary cardiomyocytes from SHAM and MI mice, we obtained cardiosomes from these cells and we incubated these cardiosomes with fibroblasts isolated from SHAM mice for 72 h. Strikingly, this procedure led to a significant increase in the abundance of miR-195 in fibroblasts treated with cardiosomes from MI donor cells but not when fibroblasts were incubated with cardiosomes from SHAM cells (Figure 3A,B); a similar response was observed when measuring the mRNA expression of genes activated in myofibroblasts, including αSMA, collagen, fibroblast activation protein (FAP), periostin, the spliced isoform of fibronectin containing extra domain A (Fibronectin ED-A) (Figure 3C–G). Similarly, the expression of genes activated during inflammatory responses, including neutrophil-recruiting chemokine (C-X-C motif) ligand 1 (CXCL1) and interleukin-6 (IL-6) was significantly augmented in fibroblasts incubated with MI cardiosomes (Figure 3H).

### 2.3. Fibroblasts Are Activated when Cultured in Conditioned Medium of Post-MI Cardiomyocytes

**Figure 4:**
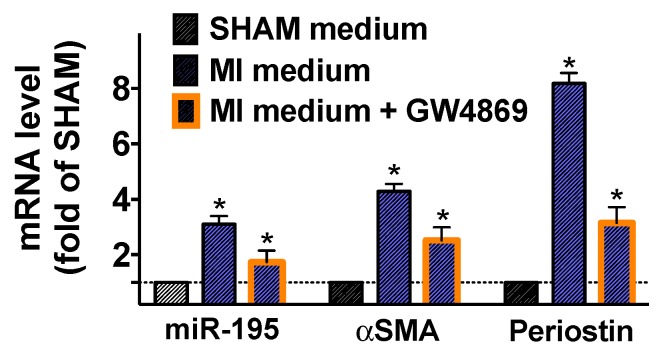
Effects of conditioned cardiomyocyte medium on the activation of fibroblasts. Conditioned medium from cardiomyocytes isolated (*n* ≥ 6 mice/group) seven days post-MI induced an upregulation of miR-195, αSMA, and periostin. However, the addition of an inhibitor of cardiosome release (GW4869 10 μM for 12 h) significantly attenuated these responses. Mean ± S.E.M. of at least three independent experiments; * *p* < 0.05 vs. SHAM.

To further substantiate our findings, we cultured fibroblasts from SHAM mice in the presence of the conditioned medium of cardiomyocytes isolated from SHAM and post-MI mice. Significantly increased levels of miR-195, αSMA, and periostin (Figure 4), were observed only in fibroblasts cultured in the medium from post-MI cardiomyocytes and these effects were markedly attenuated by GW4869, an inhibitor of neutral sphingomyelinase 2 (responsible for secretion of exosomes [35]). 

**Figure 5:**
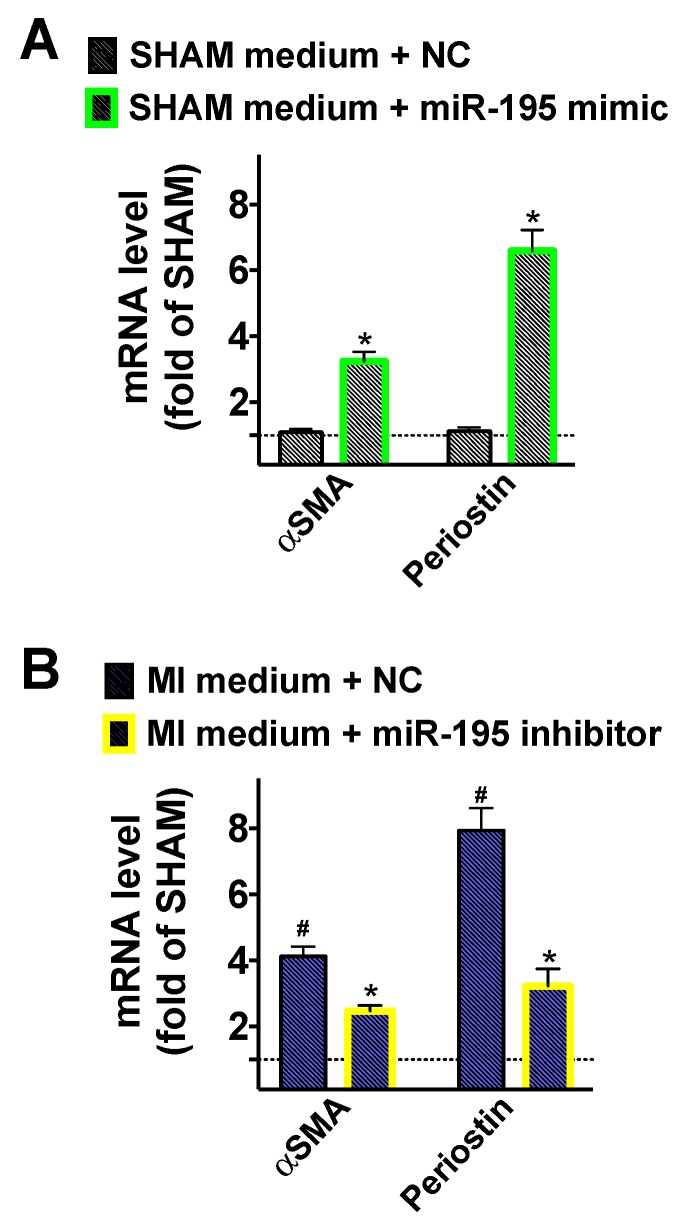
Effects of miR mimics and inhibitors on fibroblast activation. Conditioned medium from cardiomyocytes isolated (*n* ≥ 6 mice/group) seven days post-MI or SHAM surgery was added to fibroblasts transfected with miR-195 mimic (**A**), miR-195 inhibitor (**B**), or negative control miR (NC). Mean ± S.E.M. of at least three independent experiments; * *p* < 0.05 vs. SHAM medium + NC (Panel A) or vs. MI medium + NC (Panel B), # *p* < 0.05 vs. SHAM.

Moreover, we verified the mechanistic role of miR-195 in the activation of cardiac fibroblasts by using specific miR mimics (Figure 5A) and inhibitors (Figure 5B). 

## 3. Discussion

In the present study, we demonstrate for the first time that miR-195, a cardiomyocyte-specific miRNA, which is upregulated in cardiac myocytes after an ischemic insult, plays a pivotal role in the activation of myofibroblasts in a cell-cell cross-talk pathway. Indeed, our data indicate that this miRNA is secreted by injured cardiomyocytes within cardiosomes and transferred to fibroblasts, where it relieves the SMAD7-mediated inhibition of αSMA transcription, eventually leading to myofibroblast phenoconversion. The regulation of αSMA, particularly at the transcriptional level, has been the focus of recent studies and a number of transcriptional factors have been found to modulate its expression, including SMADs, myocardin-related transcription factor A/B (MRTF-A/B), CCAAT/enhancer-binding protein β (C/EBP-β), Krüppel-like factor 4 (KLF4), peroxisome proliferator-activated receptor γ (PPARγ), and NK2 homeobox 5 (Nkx2.5) [17]. We validated the interaction between this miR-195 and the 3′ untranslated region (UTR) of SMAD7 via luciferase assays. It is notable that such molecular targeting was predicted by bioinformatic approaches in both the human and murine genome.

Our results are consistent with previous investigations showing the importance of intramyocardial fibroblast-myocyte communication [36], although most of these studies focused on molecules produced by fibroblasts that regulate cardiomyocyte function [16,37,38]. Intriguingly, miR-195 has been implied in the pathophysiology of heart failure [39] and could play a role in the (dys)regulation of adrenergic-mediated responses, known to be critical in failing heart where they regulate both fibrosis and inflammation [40,41,42,43,44,45,46]: for instance, miR-195 has been reported to be upregulated by isoproterenol in the heart during catecholamine-induced hypertrophy [47]. Moreover, our findings pave the way for future investigation on the potential role of cardiosomal miRNAs in long-distance communications, since extracellular vesicles enriched in miRNAs have been recently found in blood [48,49]. Indeed, cells need to communicate efficiently with each other in order to propagate signals and coordinate functions [22].

Cardiosomes are members of the family of bioactive extracellular vesicles, lipid membrane-bound structures actively secreted or shed from multiple cell types [50,51]. This family encompasses exosomes (named cardiosomes when generated by cardiomyocytes), ectosomes, apoptotic bodies, oncosomes, shedding vesicles, microvesicles, and microparticles [52]. Since their discovery in 1983 [53], and initially regarded as membrane debris with no biological relevance, extracellular vesicles have recently obtained interest. In fact, growing attention has focused on the theory that extracellular vesicles may serve as an exquisite form of intercellular communication [23,50]: indeed, they exert key functions via transferring to recipient cells their bioactive cargos, which are different in both quantitative and qualitative terms depending on the status of the parent cell, for instance in response to ischemic injury [54,55,56,57,58,59,60,61]. Although size is often used to classify subtypes of extracellular vesicles, there is no consensus so far on a strict cut-off. Therefore, implementing a recommendation stated in a recent Position Paper of the European Society of Cardiology [62], we are herein referring to these vesicles simply as cardiosomes.

Our experiments mechanistically demonstrate that two cardiosomal miRNAs are crucial in the activation of cardiac myofibroblasts, evaluated in terms of αSMA and periostin expression. Additionally, we show that post-MI cardiosomes can trigger in cardiac fibroblasts the transcription of collagen, FAP, and fibronectin ED-A (the isoform of fibronectin produced by alternative splicing, commonly observed in activated fibroblasts). Equally important, a pro-inflammatory transcriptional activity (with augmented levels of CXCL1 and IL-6) was induced in fibroblasts by post-MI cardiosomes. Having obtained cardiosomes ex vivo, from primary isolated cardiomyocytes, is proving the exact source of such vesicles. Also, when inhibiting the release of cardiosomes using GW4869, the transcriptional regulation of αSMA and periostin was not completely blunted, suggesting that other mechanisms, in addition to cardiosomal miRNAs, are involved in myofibroblast activation. Further studies will appraise the translational potential of our findings in order to produce cardiosome-mediated therapeutic approaches to attenuate excessive post-ischemic cardiac fibrosis.

## 4. Materials and Methods

### 4.1. Animal Studies

Methods used in this paper adhere to the NIH Guide for the Care and Use of Laboratory Animals (http://oacu.od.nih.gov/regs/guide/guide.pdf). All procedures were approved by the Einstein Institutional Animal Care and Use Committee (protocol #20170411 approved on 18 August 2017). Myocardial infarction was obtained in ~6-month-old mice as follows: a small (~1 cm) thoracotomy was performed via the fourth intercostal space and the lungs retracted to expose the heart. The left anterior descending (LAD) coronary artery was located and ligated near its origin between the pulmonary outflow tract and the edge of the left atrium. Then, the chest wall was quickly closed in layers using a 3-0 suture, and animals were observed and monitored until recovery. A group of age-matched littermates undergoing sham ligation had a similar surgical procedure without tightening the suture around the artery. To estimate myocardial infarct size, serum concentrations of troponin I were measured 1 d after coronary artery ligation via enzyme-linked immunosorbent assay (ELISA, Lifespan Biosciences, Seattle, WA, USA). Transthoracic ultrasound analysis was performed in anesthetized mice (isoflurane: 5% induction, 1.5% maintenance), as previously described [34,63,64].

### 4.2. Isolation of Cardiomyocytes and Fibroblasts

Each heart was retroperfused for 20 min with the cardiac cell isolation buffer (in mM: NaCl: 113, KCl: 4.7, NaH_2_PO_4_: 0.6, KH_2_PO_4_: 0.6, HEPES: 10, Glucose: 7, Taurine: 15, 2,3-butanedione monoxime: 10, MgCl_2_: 1.2; 37 °C, pH 7.8) plus 5 mM EDTA; then, each heart was retroperfused for 30 min with the cardiac cell isolation buffer plus collagenase II (Worthington, 100 U/mL). Following the perfusion steps, the left ventricle was dissected and placed in a dish containing the cardiac cell isolation buffer plus collagenase II (100 U/mL); in this dish, ~1 mm^3^ pieces were gently dissociated from the left ventricle using two forceps. After 10 min, cardiac cell isolation buffer plus sterile, exosome-free FBS (5%) was added to the dish in order to stop the digestion and the cell suspension was passed through a 100-μm pore-size filter to remove undigested debris; then, cells were allowed to settle by gravity in 15 mL tubes for ~20 min during which a progressive Ca^2+^ reintroduction was performed in four steps (every five minutes) to reach the following concentrations: 0.25, 0.5, 0.75, and 1 mM. Sterile conditions were maintained throughout. Rod-shaped cardiomyocytes settled to a pellet and were collected, while fibroblasts and other non-myocyte cells remained in suspension. These dispersed cells were plated in 10 cm culture dishes for ~80 min: non-myocyte cells that remained attached to the dishes were allowed to grow to confluence, then trypsinized and passaged at 1:3. The characteristic morphologic appearance of the cells was confirmed at the microscope. Transfection of miR-195 mimics/inhibitors as well as of the negative control was performed following the manufacturer’s instructions.

### 4.3. Luciferase Assay

A luciferase reporter comprising the 3′ untranslated region (UTR) segment containing predicted miRNA interaction sites was used to analyze the effects of miR-195 on SMAD7 gene transcription in C2C12 cells. Cells were transfected with the 3′UTR reporter plasmid (0.05 μg) and miRNA mimics (50 nM); *Caenorhabditis elegans* cel-mir-39 mimic was used as negative control. Reagents for these experiments were purchased from ATCC (Manassas, VA, USA), GeneCopoeia (Rockville, MD, USA), Abm (Richmond, BC, Canada), and Promega (Madison, WI, USA).

### 4.4. Exosome Isolation

Exosomes were isolated by serial centrifugation as previously described [64,65]. Briefly, the culture media (depleted from serum-exosomes) [64] of primary cardiomyocytes were collected and centrifuged first at 300× *g* for 3 min to pellet cells and then at 2000× *g* for 10 min to discard dead cells. Supernatants were centrifuged at 10,000× *g* for 30 min to remove cell debris. Exosomes were then isolated from the supernatant by ultracentrifugation at 100,000× *g* for 70 min (Beckman SW28 rotor). The pellet containing the exosomes was washed with PBS and re-centrifuged at 100,000× *g* for 2 h. The number of exosomes added to the recipient cells was estimated via measurement of the esterase activity associated with exosomes (FluoroCet). According to these measurements, the recipient cells were exposed to exosome concentrations of 8 × 10^10^/mL, within the same range as those previously reported in the plasma [66]. 

### 4.5. Quantitative Real-Time PCR (RT-qPCR) and Normalization

Levels of miR-195 and miR-126 in cardiosomes were measured using individual TaqMan microRNA assays according to the manufacturer’s instructions. We quantified absolute levels of these miRs through standard curves generated using a dilution series of known input amounts of synthetic oligonucleotide. Raw PCR data (Ct values) were transformed into miR copy numbers (absolute expression) per RT reaction by using the 7300 System Sequence Detection Software. A further normalization was obtained using the exogenous UniSP6 RNA spike-in oligonucleotide and assaying for the synthetic spike-in *Caenorhabditis elegans* oligonucleotide cel-miR-39. Cellular expression of αSMA, collagen I, collagen III, FAP, fibronectin, IL-6, and periostin was determined through means of an AbiPRISM 7300 fast real-time cycler using the power SYBR Green real-time PCR master mix kit and quantified by built-in SYBR Green Analysis, as described [67]. For all non-miRNA probes, the relative amount of specific mRNA was normalized to endogenous glyceraldehyde 3-phosphate dehydrogenase (GAPDH) [68]. All RT-qPCR experiments were performed with three different wells/RNA samples (technical replicates) and each experiment was repeated at least three times (biological replicates). Sequences of oligonucleotide primers (Merck KGaA, Darmstadt Germany) for gene analysis are indicated in Supplementary Table S1.

### 4.6. Bioinformatic Analyses

We used online target prediction tools, including TarBase v.8.0, Targetscan v.7.2, and miRDB [69,70], which, by applying advanced cross referencing systems, predict biological targets of miRNAs by searching for the presence of conserved 8mer and 7mer sites that match the seed region of miRNA. 

### 4.7. Statistical Analysis

Data are expressed as means ± S.E.M. (standard error of the mean). Statistical significance was tested using the nonparametric Mann–Whitney U test or two-way ANOVA followed by Tukey-Kramer multiple comparison test, as appropriate. Significant differences were established at a *p*-value < 0.05.

## Supplementary Materials

The following are available online at https://www.mdpi.com/1422-0067/21/1/201/s1.

## Abbreviations

α-SMA	α-smooth muscle actin
CXCL1	chemokine (C-X-C motif) ligand 1
FAP	fibroblast activation protein
Fibronectin ED-A	fibronectin containing extra domain A
GAPDH	glyceraldehyde 3-phosphate dehydrogenase
IL-6	interleukin-6
MI	myocardial infarction
miRs (miRNAs)	microRNAs
Smad7	SMAD family member
