# Supplementary material for: Cardiosomal microRNAs Are Essential in Post-Infarction Myofibroblast Phenoconversion
Source: Int J Mol Sci. 2019 Dec 27;21(1):201. doi: 10.3390/ijms21010201 (PMC6982041; doi:10.3390/ijms21010201)
Supplement: Supplementary file 1 [file ijms-21-00201-s001.pdf]

Table S1. Sequences of oligonucleotide primers (*mus musculus*) and product sizes in base pairs (bp)

Gene	Forward	Reverse	bp
**αSMA**	TGTGCTGGACTCTGGAGATG	GAAGGAATAGCCACGCTCAG	148
**Collagen I**	AGCACGTCTGGTTTGGAGAG	GACATTAGGCGCAGGAAGGT	112
**Collagen III**	TGTGGACATTGGCCCTGTT	TGGTCACTTGCACTGGTTGA	117
**CXCL1**	GGTGAGGACATGTGTGGGAG	ACACGTGCGTGTTGACCATA	101
**FAP**	CGGGAAGCAACTCATGTCCT	TGATTCTCACTGCACAGCGT	106
**Fibronectin ED-A**	ACAGGGTGACCTACTCGAGC	GACTGTGTACTCAGACCCCG	116
**GAPDH**	CACTGAGCATCTCCCTCACA	TGGGTGCAGCGAACTTTAT	111
**IL-6**	AGCCAGAGTCCTTCAGAGAGA	GGAGAGCATTGGAAATTGGGG	107
**Periostin**	ACTGCTTCAGGGAGACACAC	GTCTGGCCTCTGGGTTTTCA	111
**Smad7**	GGATGGGTACATGGATGAGG	CCCAGGAGAAAGCAGACTTG	142

αSMA: α smooth muscle actin; CXCL1: chemokine (C-X-C motif) ligand 1; FAP: fibroblast activation protein; Fibronectin ED-A: fibronectin containing extra domain A; GAPDH: glyceraldehyde 3-phosphate dehydrogenase; IL-6: interleukin-6; Smad7: mothers against decapentaplegic homolog (SMAD) family member 7.
